# Myc-Induced Liver Tumors in Transgenic Zebrafish Can Regress in *tp53* Null Mutation

**DOI:** 10.1371/journal.pone.0117249

**Published:** 2015-01-22

**Authors:** Lili Sun, Anh Tuan Nguyen, Jan M. Spitsbergen, Zhiyuan Gong

**Affiliations:** 1 Department of Biological Sciences, National University of Singapore, Singapore, Singapore; 2 Department of Microbiology and Marine and Freshwater Biomedical Sciences Center, Oregon State University, Corvallis, Oregon, United States of America; University of Hong Kong, HONG KONG

## Abstract

Hepatocellular carcinoma (HCC) is currently one of the top lethal cancers with an increasing trend. Deregulation of MYC in HCC is frequently detected and always correlated with poor prognosis. As the zebrafish genome contains two differentially expressed zebrafish *myc* orthologs, *myca* and *mycb*, it remains unclear about the oncogenicity of the two zebrafish myc genes. In the present study, we developed two transgenic zebrafish lines to over-express *myca* and *mycb* respectively in the liver using a mifepristone-inducible system and found that both myc genes were oncogenic. Moreover, the transgenic expression of *myca* in hepatocytes caused robust liver tumors with several distinct phenotypes of variable severity. ~5% of *myca* transgenic fish developing multinodular HCC with cirrhosis after 8 months of induced *myca* expression. Apoptosis was also observed with *myca* expression; introduction of homozygous *tp53^-/-^* mutation into the *myca* transgenic fish reduced apoptosis and accelerated tumor progression. The malignant status of hepatocytes was dependent on continued expression of *myca*; withdrawal of the mifepristone inducer resulted in a rapid regression of liver tumors, and the tumor regression occurred even in the *tp53^-/-^* mutation background. Thus, our data demonstrated the robust oncogenicity of zebrafish *myca* and the requirement of sustained Myc overexpression for maintenance of the liver tumor phenotype in this transgenic model. Furthermore, tumor regression is independent of the function of Tp53.

## Introduction

Hepatocellular carcinoma (HCC), malignancy of hepatocytes, is the most common primary liver cancer in Central/Southeast Asia and sub-Saharan Africa [1]. As a deadly tumor with the traits of late-stage diagnosis, poor therapeutic response and bleak prognosis, it is a research hot spot for oncologists and other scientists. In humans, HCC is associated with multiple risk factors, such as hepatitis virus infection, aflatoxin, alcohol abuse, and non-alcoholic steatohepatitis, which ultimately increase genome instability and transform hepatocytes into a neoplastic state by cumulative mutations. Myc, a transcription factor which is estimated to regulate the expression of about 15% of cellular genes, is well known for its participation in many malignant conversions [2]. *MYC* gene amplification has also been frequently detected in human HCC and is especially related to advanced HCC cases [3, 4].

To understand the fundamental mechanisms underlying cancer for developing effective therapies, it is important to investigate tumor biology in animal models [5]. The zebrafish (*Danio rerio*) has now emerged as a promising animal model for cancers because of its small size, high fertility, well developed experimental resources and tools, and low maintenance costs [6]. As vertebrate species, both zebrafish and human have many conserved anatomical structures and homologous organs with similar physiological functions. Zebrafish can develop a wide spectrum of tumors in almost every tissue, which greatly resemble human malignancies in both histological characteristics and gene expression profiles [7–10]. Our previous study revealed that transgenic expression of mouse *Myc* in zebrafish lead to liver tumors [11] and another study also reported liver hyperplasia in medaka caused by transgenic expression of a quite divergent medaka myc gene [12]; however, so far no study has documented the oncogenicity of zebrafish *myc*. Due to the whole genome duplication occurred during fish evolution following divergence of the teleost and tetrapod lineages, the zebrafish genome contains two *myc* genes orthologous to human *MYC*, i.e. *myca* and *mycb* [13]. In this study we generated two zebrafish transgenic lines with inducible *myca* and *mycb* expression, respectively, and found that both *myc* paralogs were oncogenic in hepatocytes. Especially, overexpression of *myca* resulted in high grade HCC with histological traits similar to human HCC cases and introducing *tp53* null mutation to the transgenic fish accelerated liver tumor progression. Furthermore, the tumor status was addicted to constant overexpression of *myca* and suppression of transgenic *myca* expression by removal of the chemical inducer resulted in rapid tumor regression even in the *tp53^-/-^* background.

## Results

### Both *myca* and *mycb* are oncogenic

To investigate the oncogenicity of the two zebrafish *myc* paralogs, two effector transgenic zebrafish lines carrying GFP fused *myca* and *mycb*, respectively, were generated. They were then crossed with the liver-driver line [14] to obtain two double transgenic lines, named mycAG and mycBG respectively for GFP fused myca and mycb. The constructs used for these transgenic lines are illustrated in S1 Fig. and the transgenic system has been described previously for liver-specifically inducible *kras^v12^* expression [14] based on the mifepristone inducible LexPR system [15].

**Figure 1:**
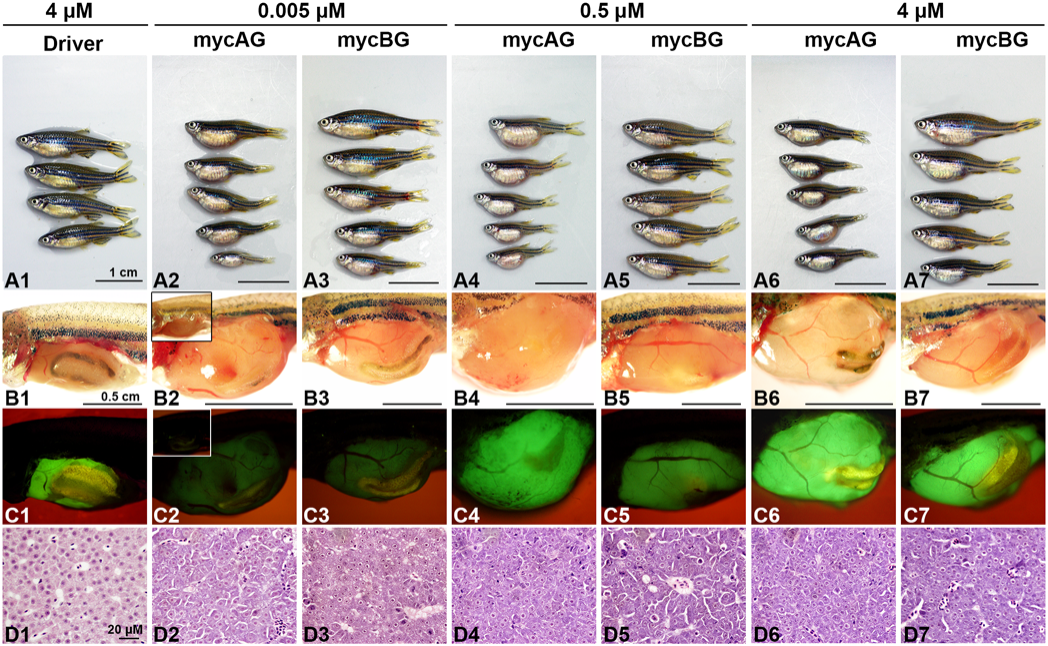
Liver tumor progression in mycAG and mycBG fish. MycAG fish and mycBG fish were induced with mifepristone of increasing concentrations, as indicated at the top of the figure, from 1 mpf and sacrificed at 2 mpi (3 mpf). (A1–A7) Exterior observation of fish from each treatment group. (B1–B7) Gross observation of liver tumors after removal of abdominal wall. (C1 to C7) The same views as those in (B1–B7) for observation of GFP expression that illustrate the shape of livers. In B2 and C2, images from uninduced mycAG fish are included as insets for comparison and there were no enlarged liver (B2) and no visible GFP expression in the liver although green fluorescent signals was observed in the gut in some fish (C2). (D1–D7) H&E staining of liver sections.

To examine the effects of myc gene expression, mycAG and mycBG fish were induced with mifepristone of different concentrations from 1 month postfertilization (mpf) and sacrificed at 2 month post-induction (mpi) or 3 mpf. The expression of transgenic *mycAG* and *mycBG* was increased with mifepristone concentrations, as manifested by the increased GFP fluorescence in Fig. 1C2–C7 as well as by RT-qPCR (S2A Fig.). Moreover, the overexpression of mycAG and mycBG resulted in overgrowth of liver (Fig. 1, B2–B7), as illustrated by GFP expression (Fig. 1, C2–C7) and also as evident from the enlarged belly (Fig. 1, A2–A7), compared to the liver in the Driver control that had only GFP expression in the liver (Fig. 1, A1, B1, C1 and S1 Fig.). Significant liver overgrowth was observed even in the case of weak expression with 0.005 μM mifepristone. Notably, the tumor size of the mycAG transgenic fish was significantly larger than that of the mycBG fish while the body length was much smaller, indicating a higher tumor burden in the mycAG fish.

Histological examination of mifepristone-induced Driver control fish showed that GFP expression has no impact on normal liver architecture and histology (Fig. 1, D1). In contrast, the liver of mycAG fish distinguished itself from normal histology with basophilic cytoplasm and enlarged eosinophilic nucleoli (Fig. 1, D2, D4 and D6). With increasing mifepristone concentrations, the liver lesions in mycAG fish progressed from hyperplasia to hepatocellular adenoma. In comparison, the tumor progression in mycBG fish was slow. At 0.005 μM mifepristone, increased mitosis was observed in mycBG fish. Although aberrant nuclei were observed, the hepatocytes retained eosinophilic cytoplasm and two-cell-thick plate organization was similar to that observed in the Driver control (Fig. 1, D3). However, the nucleus abnormality was increased with mifepristone concentration. At 4 μM mifepristone induction, the mycBG fish displayed liver hyperplasia (Fig. 1, D7) similar to that of the mycAG fish at 0.005 μM induction (Fig. 1, D2).

The above findings confirmed that both *myca* and *mycb* were oncogenic in zebrafish hepatocytes. The difference in tumor status between the two myc transgenic lines may be attributed to different levels of transgenic expression, which was confirmed by RT-qPCR analyses in S2A Fig., where transgenic *mycAG* expression was almost 5 fold of *mycBG* expression. There was a dosage-dependent induction of GFP expression with increasing mifepristone concentrations (S2C Fig.), confirmed by RT-qPCR (S2D Fig.), and noticeable increase of liver size based on 2D image measurement (S2E Fig.). This was also consistent with the generally higher GFP intensity in induced mycAG fish than mycBG fish in both fry (S2C Fig.) and adult (Fig. 1, C2–C7). Our RT-qPCR data also confirmed that both *mycAG* and *mycBG* expression were much higher than endogenous *myca* and *mycb* expression (S2A Fig.). Nuclear localization of both mycAG and mycBG fusion proteins were observed and these Myc-localized nuclei were significantly larger than these in the wild type and Driver controls, indicating pathological changes of these cells (S2B Fig.). Collectively, these observations suggest a correlation of severity of neoplasia with the level of myc mRNA expression.

### Transgenic expression of *mycAG* induces rapid liver tumors accompanied with increased proliferation and apoptosis

**Figure 2:**
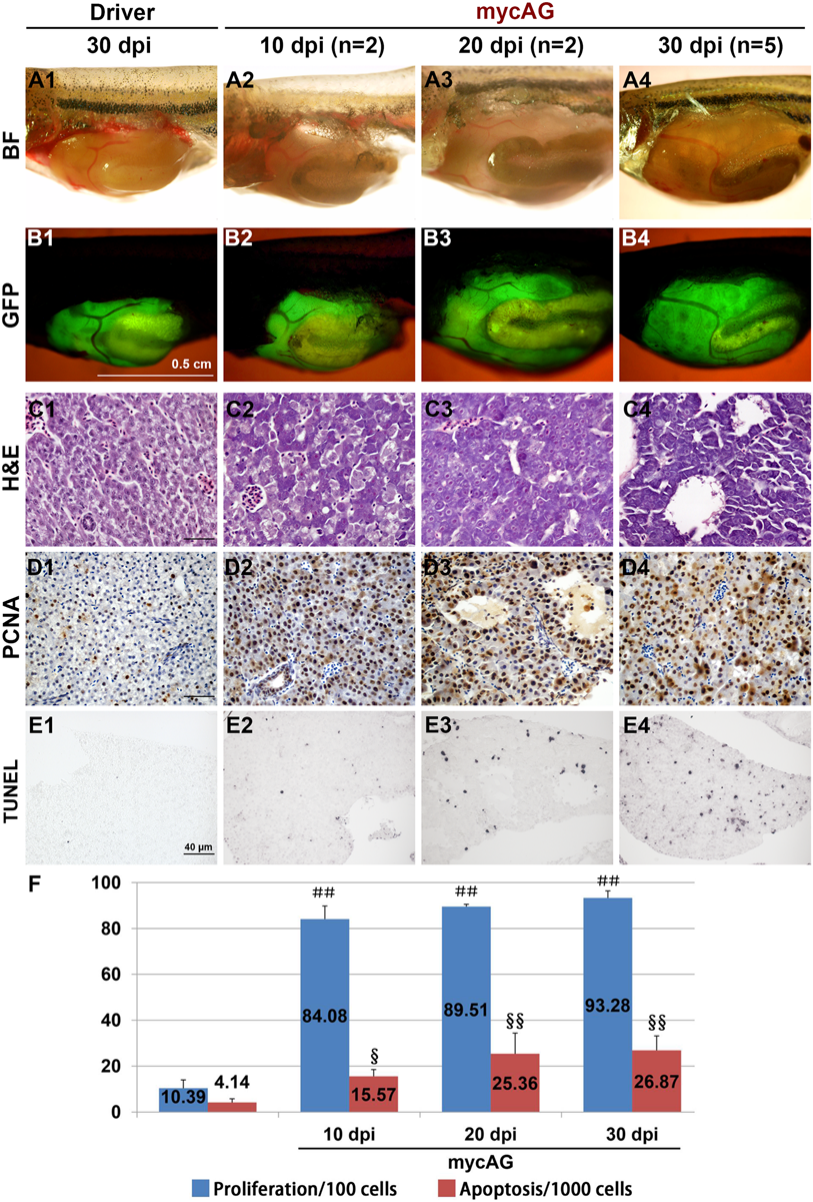
Rapid induction of live tumors by induced mycAG expression. MycAG fish were induced with 2 μM mifepristone from 1 mpf and sampled at 10, 20 and 30 dpi for gross observation as indicated in the figure. (A1–A4) Gross observation of liver tumors after removal of abdominal wall. (B1–B4) The same views as those in (A1–A4) for observation of GFP expression that illustrate the shape of livers. (C1–C4) H&E staining to show cellular alteration in the fish liver. (D1–D4) PCNA staining by immunocytochemistry to show cell proliferation. (E1–E4) TUNEL assay to reveal the apoptosis in the liver. (F) Quantitative analyses of cell proliferation and apoptosis. ＃＃ indicates highly significant difference (P<0.01) in proliferation when compared that in the Driver control at 30 dpi; § and §§, indicate significant (P<0.05) and highly significant (P<0.01) difference respectively in apoptosis when compared to that in the Driver control at 30 dpi.

As tumor progression in the mycAG line was faster and more severe than that in the mycBG line, the mycAG fish provided a more robust platform for further characterization of Myc-induced liver tumors and thus were used in the subsequent experiments. MycAG fish were induced with 2 μM mifepristone from 1 mpf and sampled at different time points. As shown in Fig. 2, A2, B2 and C2, although the liver size was still comparable with control, transformed hepatocytes with basophilic hepatocytes and distinct eosinophilic nuclei had already emerged at 10 dpi (day post-induction). From 20 dpi, all the hepatocytes had been transformed (Fig. 2, C3–C4). Moreover, the sinusoids were dilated in fast tumor progression and formed pseudoglandular phenotype with apparent ascites, as shown in Fig. 2C4 and S3 Fig.

Increased proliferation was also observed during tumor progression in the mycAG fish, as demonstrated by the PCNA staining in Fig. 2D1–D4 and 2E. Usually, apoptosis serves as a barrier in tumor progression; however, interestingly, apoptosis was also observed to increase in the liver tumors of mycAG fish, as revealed by TUNEL assay in Fig. 2E1–E4 and 2F. This is also consistent with some previous observations that Myc deregulation caused apoptosis [16–18] and this property may serves as a safeguard in normal conditions and impedes myc deregulation-caused tumor initiation.

### Variable levels of mycAG expression results in different types of liver tumors

**Figure 3:**
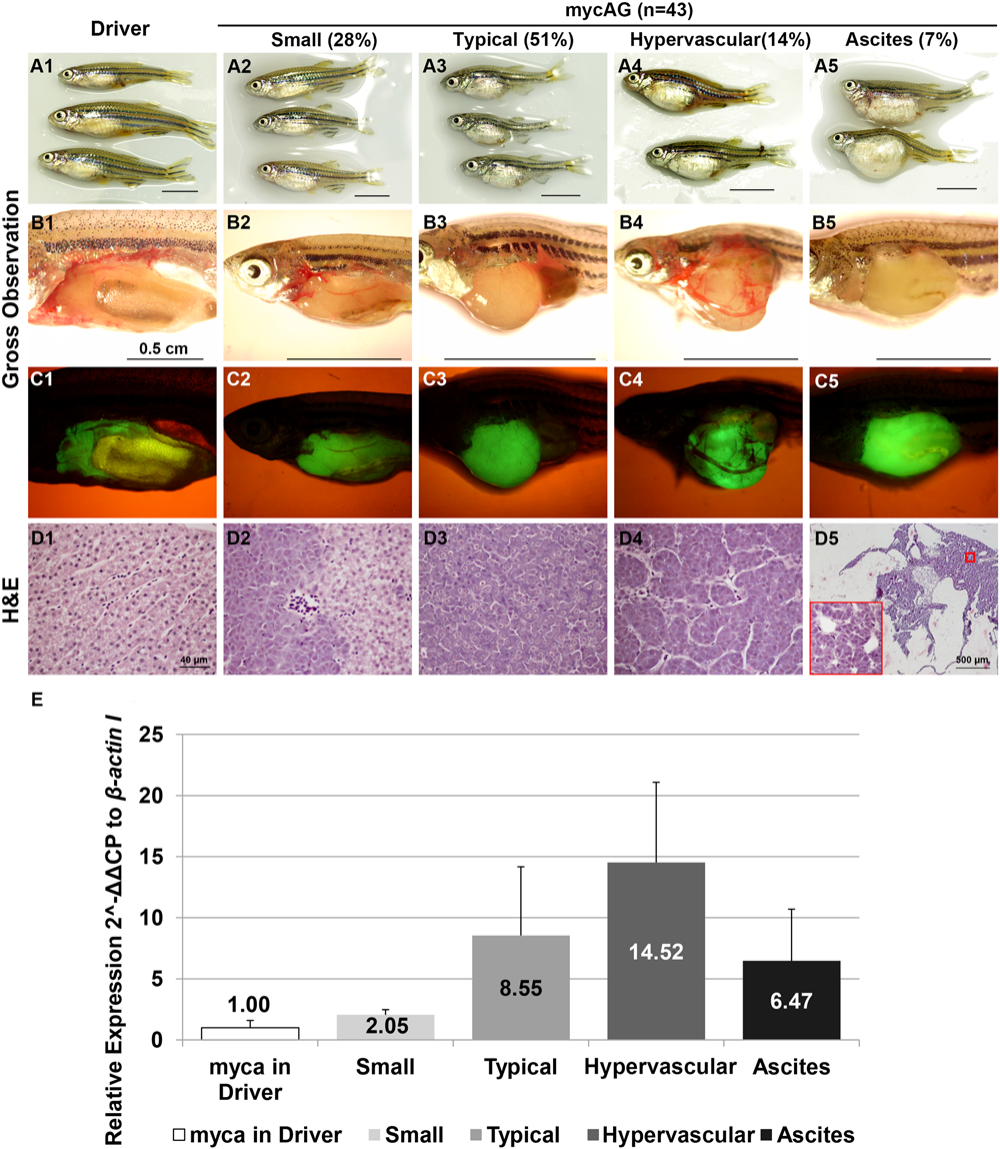
Diverse liver tumor phenotypes of mycAG zebrafish at 6 mpi. MycAG fish were induced by 2 μM mifepristone from 1 mpf and sampled at 7 mpf (6 mpi) for gross observation and histological examination. Four phenotypes were observed: Small, Typical, Hypervascular and Ascites, as indicated at the top of the figure with total numbers and percentages. (A1–A5) Exterior observation of each phenotype. (B1–B5) Gross observation of liver tumors after removal of body wall. (C1 to C5) The same views as those in (B1–B5) for observation of GFP expression that illustrate the shape of livers. (D1–D5) H&E staining of liver sections. (D1–D4) have the same magnification as indicated in scale bar in (D1). The scale bar in (D1) represents magnification for all of (D1–D4) and blow-up area in (D5). (E) Transgenic *mycAG* expression in each phenotype. Transgenic *mycAG* expression in the liver was measured by RT-qPCR and the level of expression was relative to baseline myca expression in the control Driver fish.

At 1 mpi, the majority (~87%) of induced mycAG fish showed ascites-like phenotype with yellow fluid in abdomen cavity as well as fluid-filled cysts in the liver. With the tumor progression, ascites gradually decreased in many of the mycAG fish and the relatively uniform pseudoglandular tumor type turned into divergent phenotypes. As indicated in Fig. 3, in 43 fish examined at 6 mpi/7 mpf, 28% fish showed “Small Belly” phenotype (Fig. 3A2, B2, C2), in which the tumor size was relatively small and exhibited a mixture of hepatocytes with either transformed basophilic features or near-normal eosinophilic features in histological evaluation (Fig. 3D2). 51% fish developed “Typical” tumor (Fig. 3A3, B3, C3), which were large, smooth tumors with compact tissue organization (Fig. 3D3). 14% fish displayed tumor with “Hypervascular” phenotype (Fig. 3A4, B4, C4), which displayed prominent blood vessels overgrowth (Fig. 3D4). Only 7% fish remained the “Ascites” phenotype with pseudoglandular organization of tumor cells (Fig. 3A5–D5). Interestingly, these tumor phenotypes were related to *mycAG* expression level. As shown in Fig. 3E, the “Hypervascular” tumors had the highest *mycAG* expression, followed by the “Typical” and “Ascites” phenotypes, while the “Small” phenotype had the lowest *mycAG* expression.

**Figure 4:**
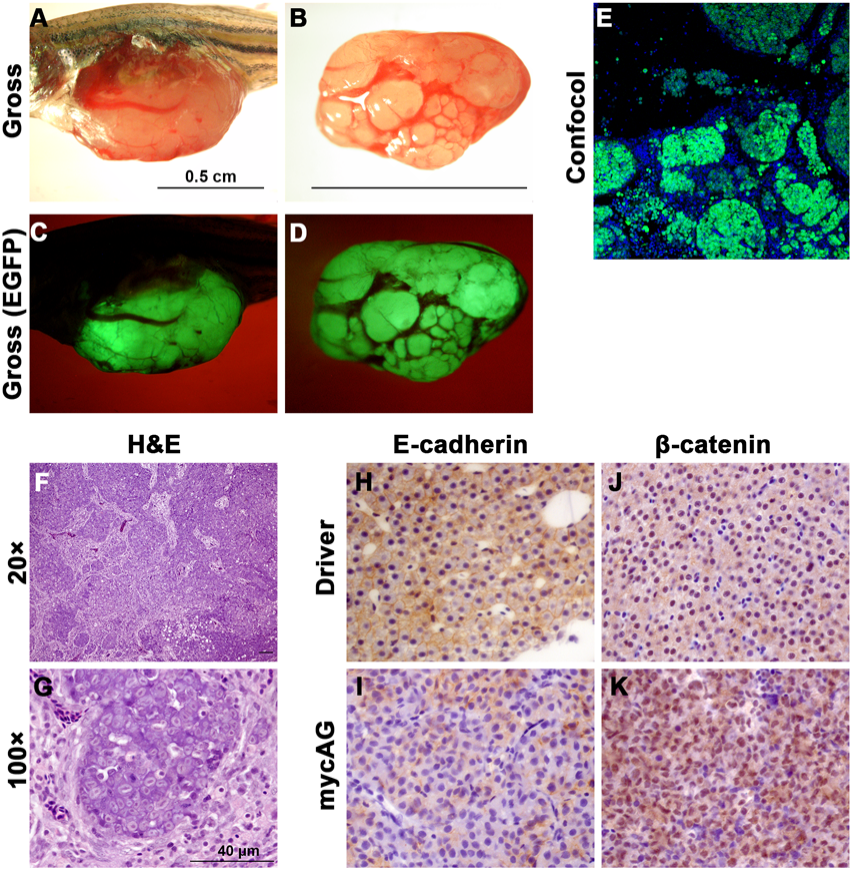
Development of multinodular HCC in late stage of mifepristone induction. MycAG fish were induced by 2 μM mifepristone from 1 mpf and sampled at 9 mpf (8 mpi) for gross observation, histological examination and immunocytochemistry. (A–D) Gross observation of multinodular HCC in two examples of mycAG fish in both bright field and GFP channel. (E) Confocal microscope image to show GFP positive hepatocytes and GFP negative cirrhosis stroma. (F,G) H&E staining of multinodular HCC sections with two different magnifications. (H,I) Immunocytochemical staining of E-cadherin in liver sections from a Driver fish (H) and a mycAG fish (I). (J,K) Immunocytochemical staining of β-catenin in liver sections from a Driver fish (J) and a mycAG fish (K).

At 8 to 9 mpi, about 5% of fish developed multinodular HCC with cirrhosis similar to that in humans (Fig. 4A–E). Although metastasis and tissue invasion were not observed in this multinodular HCC, loss of membrane-localized E-cadherin was observed (Fig. 4H,I), indicating increased motility of tumor cells and possible preparation for epithelial-mesenchymal transition. Moreover, increases of expression and nuclear translocation of β-catenin were also observed (Fig. 4J,K), suggesting that another oncogenic signaling pathway, Wnt, had been activated in the multinodular HCC.

### Homozygous *tp53^M214K^* mutation promotes tumor progression in mycAG fish

**Figure 5:**
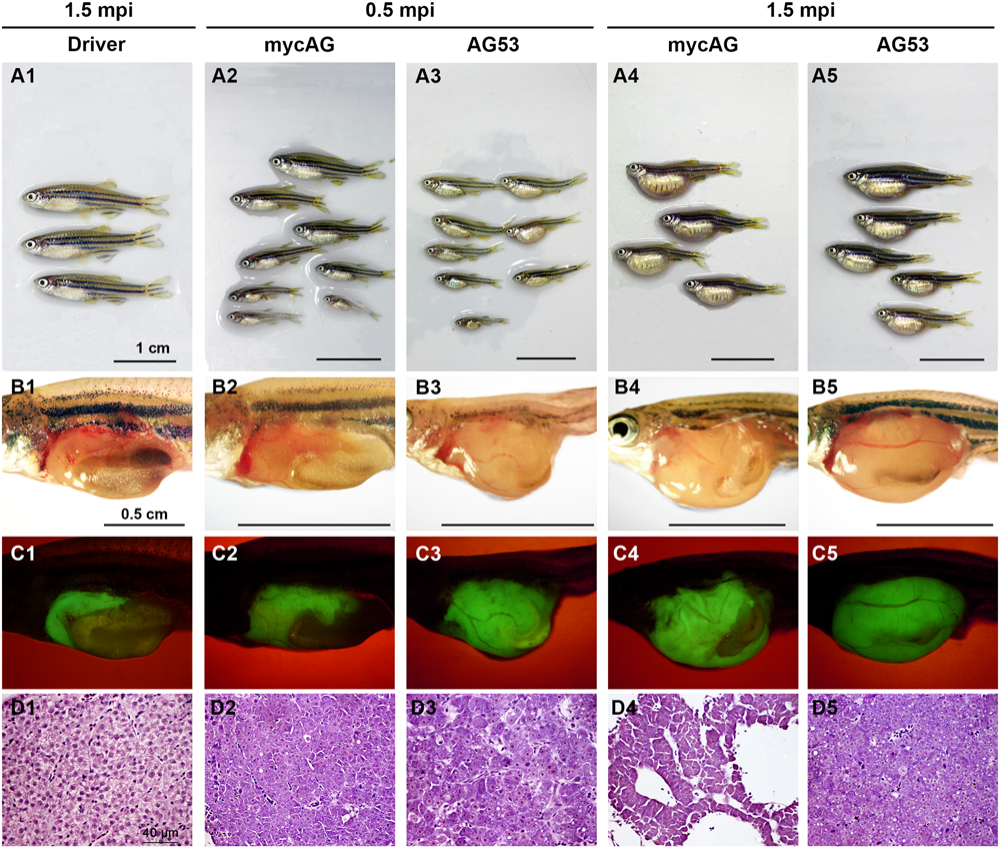
Accelerated liver tumor progression of mycAG fish in homozygous *tp53^M214K^* background (AG53). MycAG and AG53 fish were induced by 2 μM mifepristone from 1 mpf and sampled at 0.5 mpi and 1.5 mpi for gross observation and histological examination, as indicated at the top of the figure. (A1–A5) Exterior observation of each phenotype. (B1–B5) Gross observation of liver tumors after removal of body wall. (C1 to C5) The same views as those in (B1–B5) for observation of GFP expression that illustrate the shape of livers. (D1–D5) H&E staining of liver sections.

Tp53 is a major tumor suppressor genes and its mutation has been detected in a majority of human cancers [19]. To test the effect of *tp53* mutation on tumor progression in mycAG fish, we introduced a *tp53^M214K^* homozygous mutation into this mycAG transgenic fish and named this new line as AG53. Apparently, *tp53^M214K^* homozygous mutation significantly promoted tumor progression in both adult (Fig. 5) and larval stages (S4A Fig.). As shown in Fig. 5, the significant enlargement of belly of AG53 fish could be grossly observed as early as 0.5 mpi when the mycAG fish had no apparent gross changes. The body size of AG53 fish was also significantly smaller than mycAG fish, which may suggest a more serious tumor burden in AG53 fish. Differences were also manifested in histology, as shown in Fig. 5D1–D5. Although both AG53 and mycAG fish were still similar in the features of basophilic cytoplasm, enlarged nuclei and prominent eosinophilic nucleoli, the fast tumor progression in AG53 fish resulted in firm and compact tumor type rather than the pseudoglandular tumor with ascites in mycAG fish at 1.5 mpi.

To investigate the effect of *tp53^M214K^* mutation, we first examined the levels of transgenic *mycAG* and endogenous *myca* expression in the Driver control, mycAG and AG53 fish, and found both mRNAs were not affected by the mutation in AG53 fish (S4B Fig.). As many studies have demonstrated that TP53 pathway plays an important role in Myc-caused apoptosis [18], the effect of *tp53^M214K^* mutation on apoptosis was also examined. Apoptosis in the liver was significantly reduced in AG53 fish when compared to that in the mycAG fish; however, it was still higher than that in the Driver control that rarely had apoptosis signals (S4C Fig.). This observation suggested that *tp53^M214K^* mutation only blocked part of Myc-caused apoptosis, and/or that some apoptosis pathways other than Tp53 may also be activated by *myca* expression in tumor progression. Collectively, these observations suggest that the acceleration of tumor progression in *tp53^M214K^* mutation was at least partially aided by the suppression of apoptosis.

### Tumor state is dependent of sustained overexpression of transgenic mycAG

**Figure 6:**
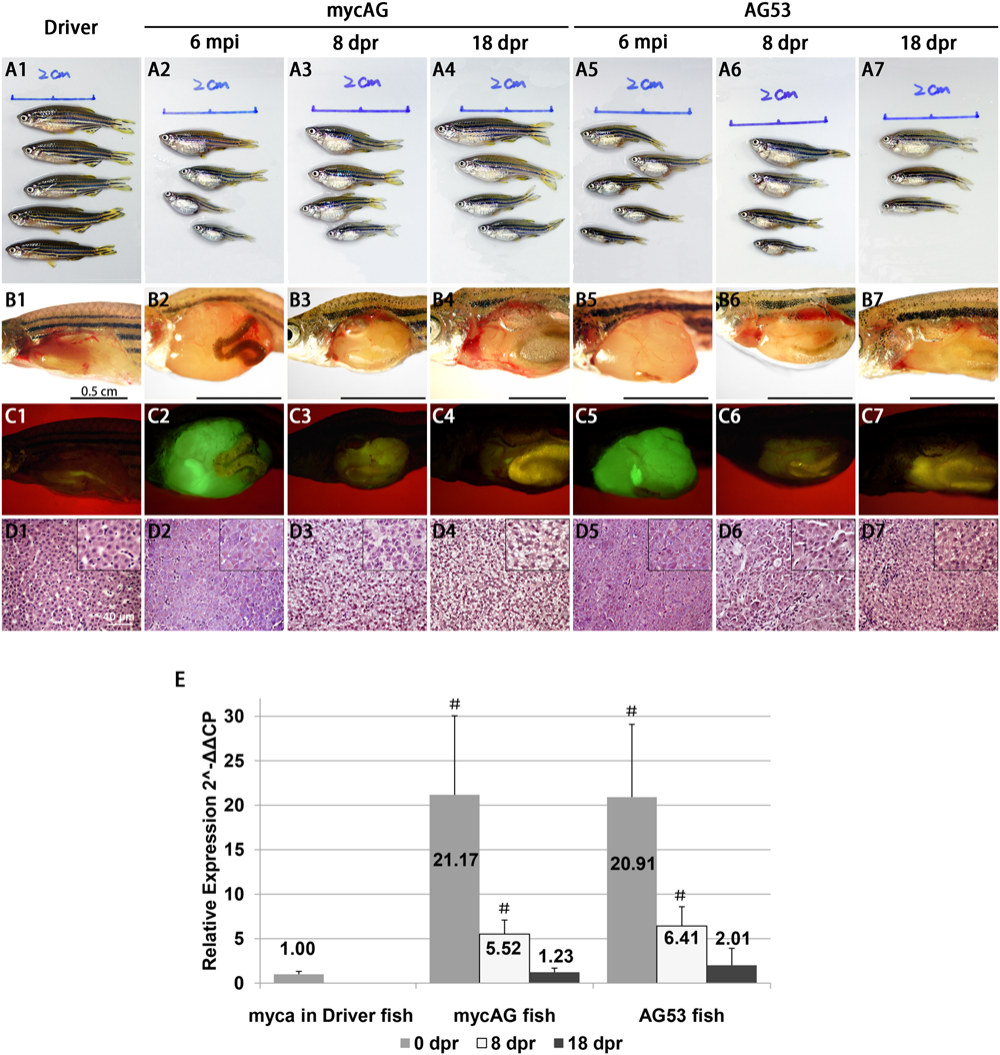
Tumor regression in mycAG fish and AG53 fish after mifepristone withdrawal. Mifepristone was withdrawn at 6 mpi (7 mpf), MycAG line and AG53 fish were sampled at 0 dpr, 8 dpr and 18 dpr for gross observation and histological examination, (A1–A7) Exterior observation of each phenotype. (B1–B7) Gross observation of liver tumors after removal of abdominal wall. (C1–C7) The same views as those in (B1–B5) for observation of GFP expression that illustrate the shape of livers. (D1–D7) H&E staining of liver sections. Larger magnifications are shown in insets. (E) MycAG mRNA level in 0-dpr, 8-dpr and 18-dpr liver samples as quantified by RT-qPCR. Each group had three biological replicates. # indicates significant difference (P<0.05) when compared with *myca* expression in the Driver.

One of the important features of the LexPR inducible expression system is the feasibility of inactivation of transgenic expression by withdrawal of mifepristone [14]. To investigate the effect of suppression of transgenic mycAG expression in both mycAG and AG53 fish, mifepristone treatment was stopped at 6 mpi/7 mpf. As shown in Fig. 6E, the mRNA expression of transgenic *mycAG* in the liver greatly decreased within 8 days of mifepristone withdrawal (8 dpr, day post-regression). At 18 dpr, *mycAG* level was comparable to the endogenous *myca* expression in the control fish liver. Consistently, GFP signal also faded away rapidly (Fig. 6, C2–C7). Only very faint GFP was observed in mycAG and AG53 fish liver at 8 dpr (Fig. 6, C3 and C5) and essentially no GFP could be detected at 18 dpr (Fig. 6, C4 and C7).

**Figure 7:**
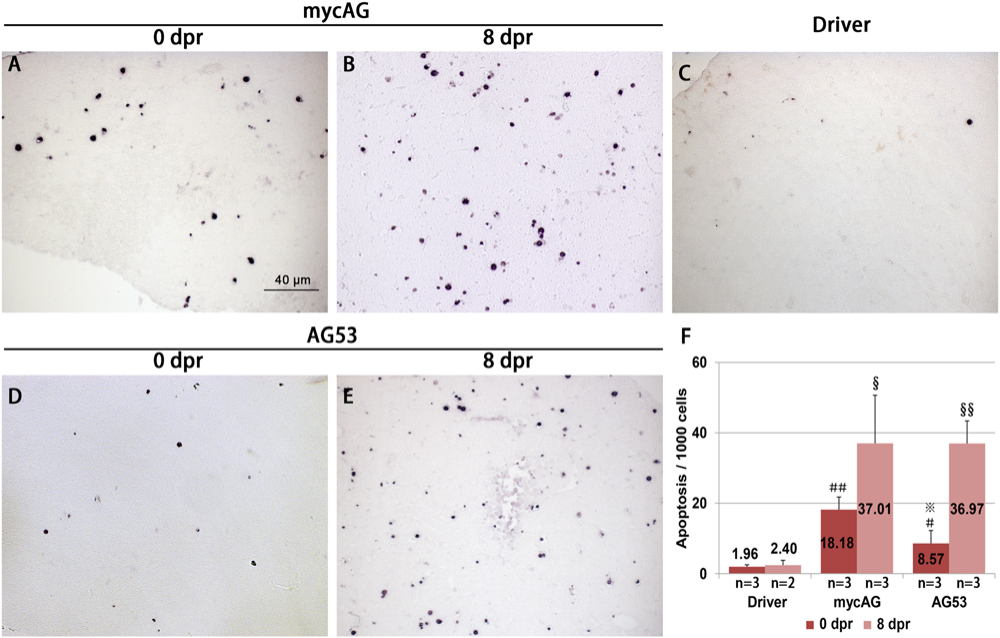
Increase of apoptosis after mifepristone withdrawal. MycAG line and AG53 fish were induced by 2 μM mifepristone from 1 mpf and mifepristone was removed after 6 month induction for tumor regression. These fish were then collected for TUNEL assay. (A–E) TUNEL assay for apoptosis on liver sections from different types of fish at different time points as indicated. dpr, day post regression. (F) Quantification of apoptotic cells in liver sectons. ＃ and ＃＃ indicate significant difference with p-values of 0.05 and 0.01 respectively when compared to Driver at 0 dpr. § and §§ indicate significant difference with p-values of 0.05 and 0.01 respectively when compared to Driver at 8 dpr. ※ indicates significant difference (P<0.05) when compared with AG at 0 dpr.

At 6 mpi, the AG53 liver tumor had a uniform tumor type with early HCC traits, while the tumor histologic features in mycAG line fish were divergent, as mentioned earlier. With the removal of mifepristone, the tumors shrunk fast in both mycAG and AG53 fish, while the body size and length increased significantly (Fig. 6, A2–A7). At the histological level, at just 8 dpr, the neoplastic hepatocytes features observed earlier had been replaced by tissue characterized by a relatively normal appearance with eosinophilic cytoplasm and basophilic nuclei (Fig. 6, D3 and D6). At 18 dpr, the appearance of the cells (Fig. 6, D4 and D7) was already similar to the Driver control (Fig. 6, D1). Moreover, cytoplasmic granules also increased in both mycAG and AG53 fish hepatocytes during tumor regression, which was a sign of restoration of hepatocyte function. Previously we have observed a rapid increase of apoptosis during liver tumor regression in our *xmrk*-induced liver tumors [20]. To examine whether apoptosis was involved in tumor regression in *tp53^M214K^* background, TUNEL assay was carried out in the regression fish. Apoptosis was rare in control livers from the Driver fish (Fig. 7C), but increased with the tumor progression in mycAG fish (Fig. 7A) and dramatically increased in the regression process (Fig. 7B and 7F). As the normal liver has a very low level of apoptotic cells and there was a decrease of GFP-labeled hepatocytes, it is likely that these apoptotic cells were mainly from transformed hepatocytes, similar to the observation in tumor regression from *xmrk* oncogene transgenic zebrafish [20]. Compared to the mycAG line fish, almost the same level of apoptosis was also observed in AG53 fish liver tumor regression (Fig. 7E and 7F). Therefore, these observations indicated that some pathways other than p53 were also involved in apoptosis in the process of tumor regression.

## Discussion

### Both Zebrafish *myca* and *mycb* are Oncogenic in Hepatocytes

It is generally accepted that vertebrates underwent two rounds of whole genome duplication during evolution from invertebrates and there was an additional round of teleost-specific whole genome duplication about 350 Ma ago [21, 22]. Phylogenetic analysis of zebrafish *myc* family also indicated that two paralogs, *myca* and *mycb*, arose from a common ancestor in the last whole genome duplication [23]. Although the expression of both of them are found in mitosis hotspots in development [13], many differences exist between the two paralogs. For example, *myca* is mainly expressed in brain while *mycb* is in lateral line neuromasts [13]. In ciliary marginal zone, *myca* and several other myc family genes are required in maintenance of continuous cell replacement; in contrast, no *mycb* expression is found in this process [24]. We also found that the *mycb* expression was much higher than *myca* in adult fish liver (S2A Fig.) and their subcellular localization might be different too (S2B Fig.). All these findings suggested that *myca* and *mycb* could have different physiological functions. However, overexpression of the two myc genes in our transgenic models indicated that both have equivalent cellular function in oncogenesis. The difference of severity of tumor induction between the two myc oncogenes are likely due to the level of induced expression as we observed an increasing severity of tumor progression from both mycAG and mycBG transgenic lines with increased mifepristone inducer, although the possibility that *myca* is more potent in ongogenesis than *mycb* could not be completely ruled out. It is also interesting to note that endogenous *mycb* expression was much lower in induced mycAG fish than those in induced mycBG and Driver fish (S1A Fig.); this could be due to a negative feedback control of endogenous myc expression by high level of transgenic myc expression.

In the present study, we also found an apparent dosage-dependent induction of transgenic myc expression, which in turn caused an increasing tumor severity (Fig. 1). A long time of induction (up to 8 months) with 2 μM mifepristone caused essentially 100% mycAG fish to grow liver tumors, among which, 5% of them had been confirmed to have multimodular HCC phenotype. The rest fish also showed some other malignant traits or signs of serious liver damage (Fig. 3). For example, ascites itself is an important clinical syndrome in late stage human liver cancer, and at the histological level, the compact hepatocytes organization in the “Typical” phenotype and neo-anginogenesis in the “Hypervascular” phenotype are also important properties in human HCC. Therefore, though not confirmed as HCC, these induced mycAG fish did show the deterioration and transition into malignant status.

### Liver tumor progression in mycAG fish is accelerated by suppression of apoptosis with *tp53^M214K^* Mutation

It seems that different levels of mycAG expression also resulted in different tumor types, although all the mycAG fish exhibited similar phenotypes at the early tumor stage (before 1 mpi), such as pseudoglandular tissue pattern and ascites (S3 and S4 Figs.). It was apparent that sustained high level expression of *myca* was necessary in the development of advanced neoplasms in later stages. Myc provoked apoptosis has been observed in many studies [18] and we also observed increased apoptosis in the liver tumor of mycAG fish (Fig. 2C1–C4). However, HCC was still developed in our model eventually, suggesting that the oncogenicity of mycAG fish is robust enough to overcome the apoptosis effect and to maintain the progression of malignant status. Moreover, it has also been found that in some tumor models, attenuating apoptosis is necessary for successful malignant transformation [25, 26]. In this project we observed that introducing a *tp53^M214K^* homozygous mutation which significantly suppressed apoptosis could accelerate liver tumor progression.

### Liver tumor regression does not require Tp53

Reversible neoplasms have been reported in antisense-treated human cancer cell lines and animal cancer models with conditional oncogene expression [27], including our previously reported transgenic zebrafish models with inducible expression of Xiphophorus *xmrk* oncogene and mouse *Myc* respectively [11, 20]. This phenomenon, i.e. malignant status dependency on sustained activation of a specific oncogene, is named “oncogene addiction” [27]. Addiction to Myc has also been reported in several previous studies. For example, in a tet-off mice acute myeloid leukemia model, introduction of doxycycline lead to suppression of transgenic *MYC* expression and caused regression of the tumor [28]. Similarly, in a Myc-induced mouse HCC model, inactivation of transgenic Myc also caused rapid tumor regression with increased apoptosis [29]. However, not all the tumors could regress after the elimination of the original oncogenic factor, especially when additional oncogenic mutations have occurred, which is essentially in all cases in human cancers. For example, the mammary adenocarcinoma in a mouse model established by *MYC* overexpression could not regress after abolishing *MYC* expression because of a secondary spontaneous activating mutation in *Kras* [30].

In the mycAG and AG53 transgenic zebrafish reported here, inactivation of mycAG expression after removal of mifepristone resulted in rapid tumor regression in both groups of fish and all the hepatocytes were reverted to a normal appearance histologically (Fig. 6). While it is anticipated for a rise of apoptosis in mycAG fish during tumor regression, which is consistent with several previous reports [20, 28], it is unusual to observe a similar increase of apoptosis in AG53 fish. *TP53* is a well-known tumor suppressor gene and a common function of Tp53 is to induce apoptosis of damaged or tumorigenic cells for elimination [19]. It has been previously reported for a Myc transgenic mouse model with hematopoietic tumors that tumor regression by inactivation of transgenic Myc requires Tp53 as only incomplete tumor regression was observed when Tp53 is lost [31]. In our AG53 fish model, although the homozygous *tp53^M214K^* mutation could largely abolish apoptosis in the tumor progression stage (S4C Fig.), but it seems that there was no effect during tumor regression. These observations suggested that the malignant status of hepatocytes is addicted to sustained overexpression of zebrafish *myca* but tumor regression does not require the presence of Tp53. Since similar levels of apoptosis were observed in both mycAg and AG53 fish during the initial stage of tumor regression, it is likely that apoptosis during tumor regression is independent of the Tp53 pathway.

## Material and Method

### Generation of mycAG, mycBG and AG53 transgenic fish

This study involving zebrafish was carried out in strict accordance with the recommendations in the Guide for the Care and Use of Laboratory Animals of the National Institutes of Health. The protocol was approved by the Institutional Animal Care and Use Committee (IACUC) of the National University of Singapore (Protocol Number: 096/12). All surgery was performed under sodium pentobarbital anesthesia, and all efforts were made to minimize suffering. The Liver-Driver line, *Tg(fabp10:LexPR; LexA:EGFP)*, was generated in a previous study [14]. Two Effector lines, *Tg(cryB:mCherry; LexA:EGFP-myca)* and *Tg(cryB:mCherry; LexA:EGFP-mycb)*, were generated in the present study using constructs depicted in S1 Fig. Both transgenic lines were identified by visualization of mCherry expression in lens under the *cryB* promoter. To enhance genome integration, the *Ac/Ds* transposon system was adopted [32], in which, a DNA construct was co-injected with *in vitro* transcribed transposase mRNA into zebrafish embryos at the 1–2 cell stage. The injected embryos were then raised for founder screening and transgenic F1 were confirmed by mCherry expression. From the F1 generation, both effector lines were maintained by crossing with the liver-driver fish and double transgenic fish were selected based on mCherry expression in the lens (Effector) and constitutive GFP expression in the liver (Driver). Multiple *myca* and *mycb* effector transgenic lines were generated and they all generated obvious liver tumors in early stages (S2F Fig.); in this study, we followed only one transgenic line each of these myc genes and the double transgenic fish were named mycAG and mycBG accordingly in this report.

The mycAG fish with *tp53^M214K^* homozygous mutation were generated by crossing mycAG fish with a *tp53^M214K^* homozygous mutant fish [33]. The heterozygous offspring were raised by incrossing and homozygosity for *tp53^M214K^* was selected by genotyping with PCR primer as indicated in S1 Table [34]. The mycAG fish were then maintained in homozygous *tp53^M214K^*, named AG53, by incrossing for experiments.

### Mifepristone treatment

Mifepristone (RU-486, Sigma-Aldrich #M8046) was first dissolved in ethanol and diluted in fish water for the final concentrations. The treatment was conducted in petri dishes or 6-L tanks at a density of ~50 larvae or ~25 adult per dish or tank, respectively. The water with mifepristone was topped up every 3–4 days and totally changed every two weeks.

### Reverse transcription-quantitative PCR (RT-qPCR)

Total RNA was extracted using TRIzol (Invitrogen #15596–018) and reverse-transcribed into cDNA. To distinguish the endogenous *myca* and *mycb* mRNAs from the transgenic *mycAG* and *mycBG* mRNAs, *myca* and *mycb* primers were targeted at the coding region and 3′ untranslated regions while *mycAG* and *mycBG* primers at the myc coding region and the GFP region. *β-actin* was used as an inner control. The primer sequences are presented in S1 Table.

### Paraffin section and histological analyses

Fish samples were fixed in 10% neutral buffered formalin solution (Sigma, #HT5012). After dehydration and embedding with paraffin, sectioning was performed using a Leica microtome, and all the samples were sectioned sagittally at a thickness of 4 μm. For ordinary observation, the samples were stained with Hematoxylin (Sigma, #51275) and Eosin (Sigma, #HT110232). For immunohistochemistry, the primary antibodies used were PCNA (AnaSpec Inc. #55421), β-catenin (Abcam, #ab32572) and E-cadherin (Abcam, #ab1416). After washes with phosphate buffered saline (PBS) with 0.1% Tween 20 (PBST) and incubation with appropriate secondary antibody from the Dako Kit set (DAKO, Denmark, #346811), color was developed by using the Liquid DAB & Substrate Chromogen system (DAKO, #346811). The slides were also conter-stained with 5 μg/ml DAPI (4′, 6-Diamidine-2′-phenylindole dihydrochloride) to facilitate nucleus observation.

### TUNEL assay

TUNEL assay was conducted with ApopTag Plus In Situ Apoptosis Fluorescein Detection Kit (Chemicon international, #S7111). After rehydration and treatment with 20 μg/ml proteinase K in PBS at room temperature, the sections were treated with TDT enzyme and incubated with alkaline phosphate-conjugated Anti-DIG. Next the slides were balanced in pH 9.5 buffer (0.1 M Tris-HCl/50 mM Mgcl2/ 10 mM NaCl/0.1% Tween 20) and finally the signal colors were developed using nitro-blue tetrazolium/5-bromo-4-chloro-3′-indolyphosphate in pH 9.5 buffer.

### Statistical analyses

T-test was used for all assays in the present studies, including cell counting in DOPI, PCNA and TUNEL staining, measurement of 2D liver size and RT-qPCR. Statistical significance was indicted in figure legends.

## Supporting Information

S1 Fig Schematic representation of DNA constructs used in the mifepristone-inducible expression system. The mifepristone inducible expression system has been described previously [14, 15]. The Driver construct consists of a chimeric LexPR transcription activator under the liver-specific *fabp10a* promoter. The driver construct also contains an EGFP effector transcription unit under the LexA operator. Two effector constructs for expression of *myca* and *mycb* under the LexA operator were also made. The effector constructs also contain an mCherry reporter gene under a lens-specific *crybb* promoter for identification of effector fish. Both the driver and effector constructs are flanked with transposon Ds elements for improving of efficiency of genome insertion [32].
(PDF)

S2 Fig Effects of *mycAG* and *mycBG* expression in zebrafish liver. (A) Expression of transgenic *mycAG* and *mycBG* in comparison with expression of endogenous *myca* and *mycb* genes. Liver RNA from 1 mpi (2 mpf) fish treated with 2 μM mifepristone were analysed by RT-qPCR and expression values are relative to the level of endogenous *myca* mRNA, which is arbitrarily set as 1. (B) Subcellular localization of mycAG and mycBG fusion proteins. Fish were all treated with 2 μM mifepristone and liver tissues were collected at 1 mpi (2 mpf) for cryosection. GFP signal was recorded by confocal microscope with the same exposure time. Nuclei were stained by DAPI and recorded in the blue channel. Wild type liver was used as a negative control. (C) Dosage-dependent effect of mifepristone induction on GFP or Myc-GFP expression and liver size in Driver, mycAG and mycBG larvae. These transgenic fish were induced with mifepristone at different concentrations from 3 dpf and photographed at 8 dpi (5 dpi). (D) Dosage-dependent increase of *mycAG* and *mycBG* expression as measured by RT-qPCR. (E) Quantification of liver size based on 2D images at 4 μM mifepristone. Size of liver of larvae was measured according to GFP signal area. # and ## indicate significant difference with p-values of 0.05 and 0.01 respectively when compared with the liver size in the Driver. (F) Induction of liver tumors from other mycAG transgenic families. Representative fish from three other mycAG transgenic families are shown with gross observations of liver tumors (upper panels) and GFP expression (lower panels) to illustrate the liver tissues as described in Fig. 1B and 1C.
(TIF)

S3 Fig Ascites in mycAG zebrafish. MycAG fish were induced by 2 μM mifepristone from 1 mpf and sampled at 7 mpf (6 mpi) for gross observation and histological examination. (A) Gross observation of the mycAG fish with ascites phenotype in monotone background. (B) Gross observation of the same fish in (A) under a dark background to show transparent belly. (C) H&E staining of the fish presented in (A,B). (D, E) Isolated liver tumor from another mycAG fish with ascites for GFP view (D) and for view in a dark background to show transparent, foam-like structure (E). Liquid was observed in cysts in the liver. (F) H&E staining of the liver tumor presented in (C,D).
(PDF)

S4 Fig Reduction of apoptosis and acceleration of liver tumor progression in mifepristone-induced mycAG fish with *tp53^M214K^* mutation (AG50). (A) Comparison of liver size between mycAG and AG53 larvae. Larvae were induced with 2 μM mifepristone from 3 dpf and photographed at 5 dpi (8 dpf). Liver size was measured based on 2D GFP images as previously described [35]. ## and # indicate significant difference with p-values of 0.01 and 0.05 respectively when compared with the liver size in the Driver53 (Driver fish in *tp53^M214K^* mutation background). §§ and § indicate significant difference with p-values of 0.01 and 0.05 respectively when compared with the liver size in the Driver. ++ indicates significant difference (P < 0.01) when compared with the liver size in AG53. (B) Induction of *mycAG* expression in mycAG and AG53 fish. Total RNA was extracted from 5 dpi/8 dpf larvae treated with 2 μM mifepristone for RT-qPCR analyses. Each group has three biological replicates and there was no significant effect on induction of *mycAG* mRNA by *tp53^M214K^* mutation. # indicates the expression differences when compared to endogenous *myca* expression in Driver fish (p < 0.05). (C) Apoptosis in AG53 and mycAG fish livers. Liver sections from 2 mpi (3 mpf) driver, mycAg and AG50 fish were used for TUNEL staining. Each group has 3 to 4 biological replicates and apoptosis was counted and represented in column. ＃＃, indicates the difference in proliferation when compared with Driver is significant (P < 0.01). §§ indicates significant difference between mycAG and AG50 fish (P < 0.01).
(PDF)

S1 Table Primer Sequences Used in PCR. (DOCX)
