# Supplementary figures and images for: Myc-Induced Liver Tumors in Transgenic Zebrafish Can Regress in tp53 Null Mutation
Source: PLoS One. 2015 Jan 22;10(1):e0117249. doi: 10.1371/journal.pone.0117249 (PMC4303426; doi:10.1371/journal.pone.0117249)

# Driver Construct

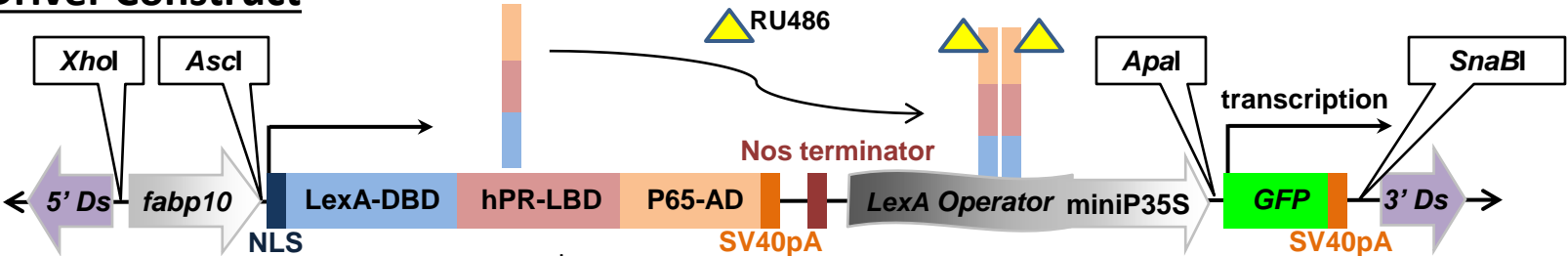

# Effector Constructs

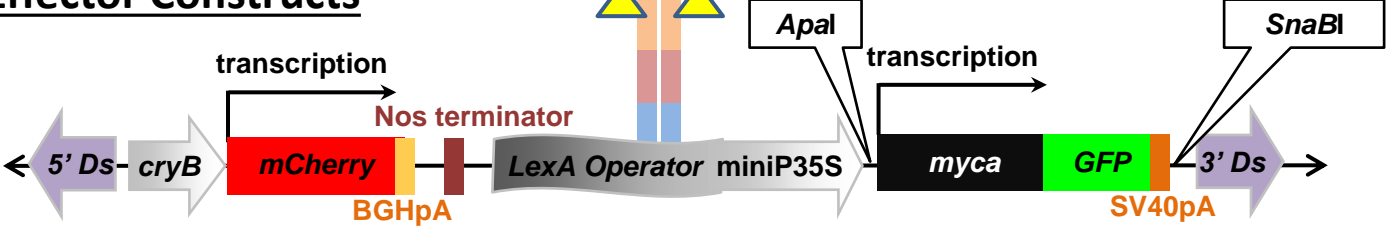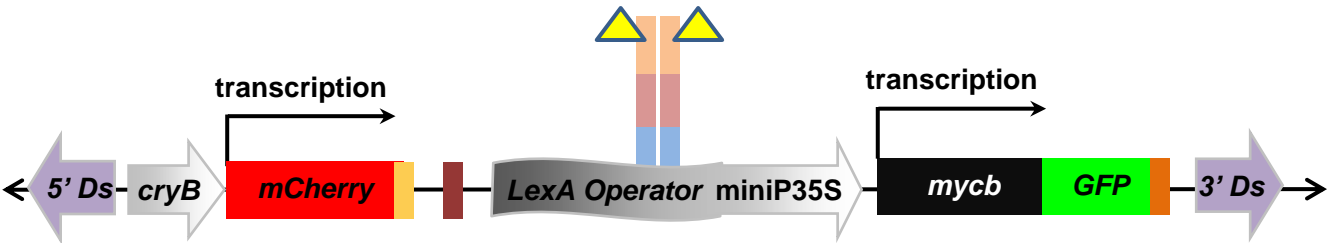

Supplement: S1 Fig — The mifepristone inducible expression system has been described previously [14, 15]. The Driver construct consists of a chimeric LexPR transcription activator under the liver-specific fabp10a promoter. The driver construct also contains an EGFP effector transcription unit under the LexA operator. Two effector constructs for expression of myca and mycb under the LexA operator were also made. The effector constructs also contain an mCherry reporter gene under a lens-specific crybb promoter for identification of effector fish. Both the driver and effector constructs are flanked with transposon Ds elements for improving of efficiency of genome insertion [32]. (PDF) [file pone.0117249.s001.pdf]

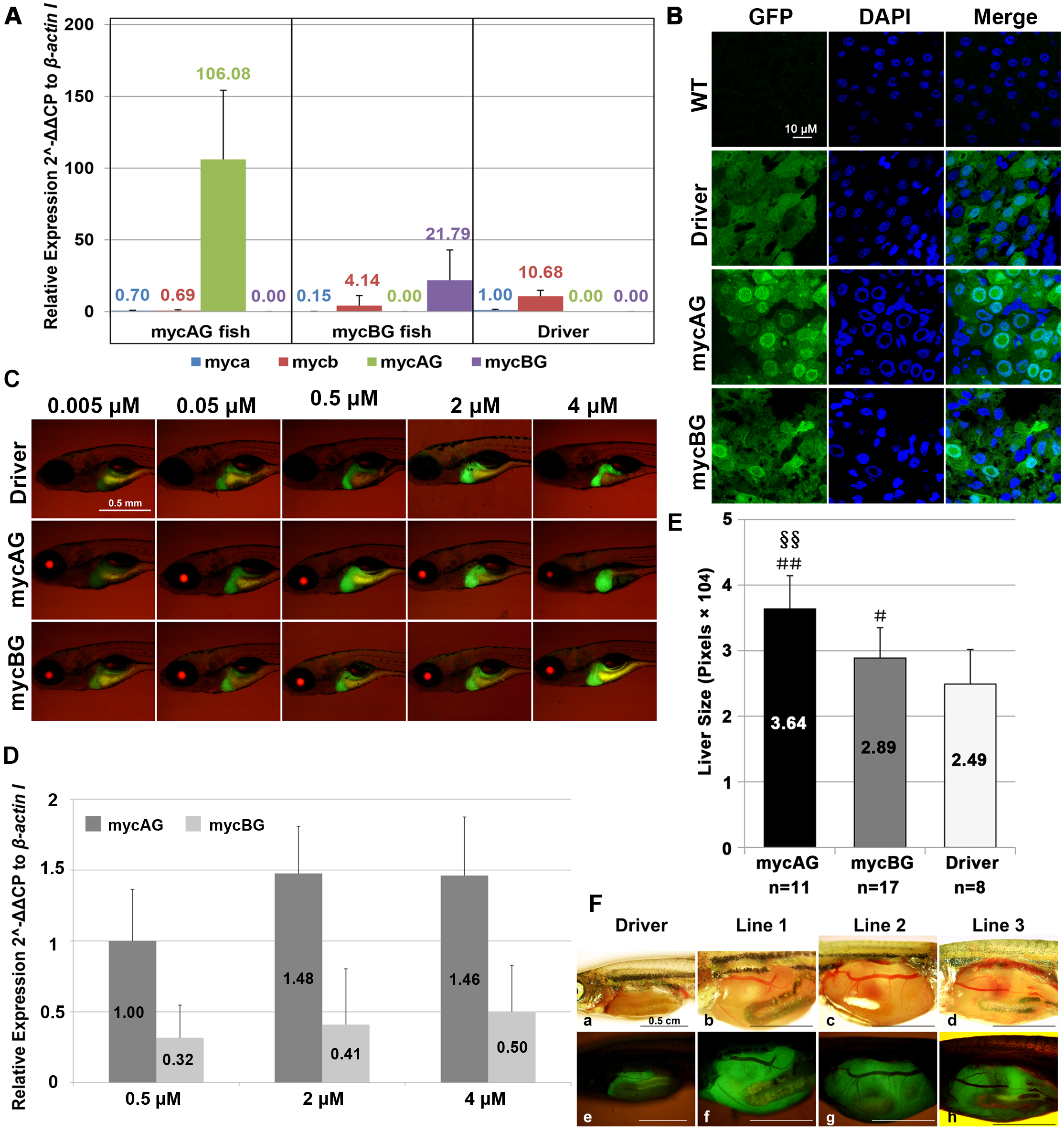

Supplement: S2 Fig — (A) Expression of transgenic mycAG and mycBG in comparison with expression of endogenous myca and mycb genes. Liver RNA from 1 mpi (2 mpf) fish treated with 2 μM mifepristone were analysed by RT-qPCR and expression values are relative to the level of endogenous myca mRNA, which is arbitrarily set as 1. (B) Subcellular localization of mycAG and mycBG fusion proteins. Fish were all treated with 2 μM mifepristone and liver tissues were collected at 1 mpi (2 mpf) for cryosection. GFP signal was recorded by confocal microscope with the same exposure time. Nuclei were stained by DAPI and recorded in the blue channel. Wild type liver was used as a negative control. (C) Dosage-dependent effect of mifepristone induction on GFP or Myc-GFP expression and liver size in Driver, mycAG and mycBG larvae. These transgenic fish were induced with mifepristone at different concentrations from 3 dpf and photographed at 8 dpi (5 dpi). (D) Dosage-dependent increase of mycAG and mycBG expression as measured by RT-qPCR. (E) Quantification of liver size based on 2D images at 4 μM mifepristone. Size of liver of larvae was measured according to GFP signal area. # and ## indicate significant difference with p-values of 0.05 and 0.01 respectively when compared with the liver size in the Driver. (F) Induction of liver tumors from other mycAG transgenic families. Representative fish from three other mycAG transgenic families are shown with gross observations of liver tumors (upper panels) and GFP expression (lower panels) to illustrate the liver tissues as described in Fig. 1B and 1C. (TIF) [file pone.0117249.s002.TIF]

## Gross Observation

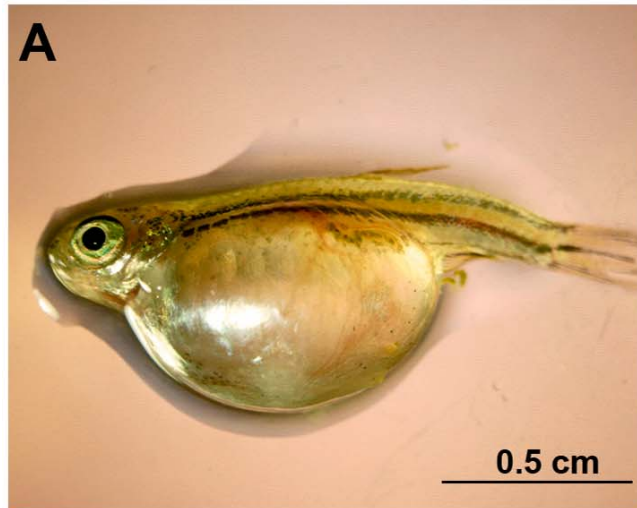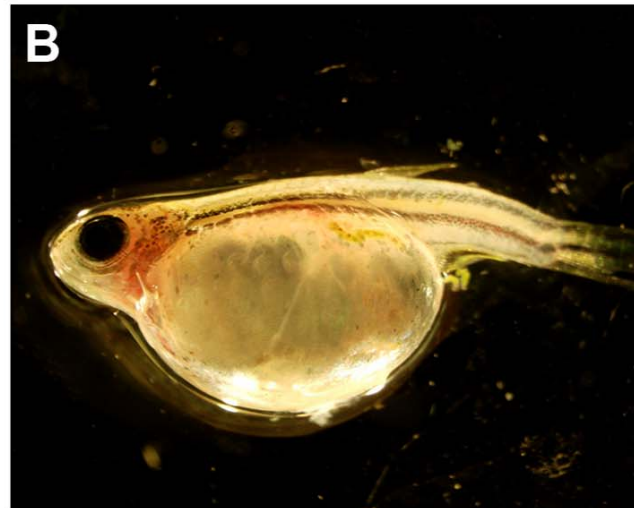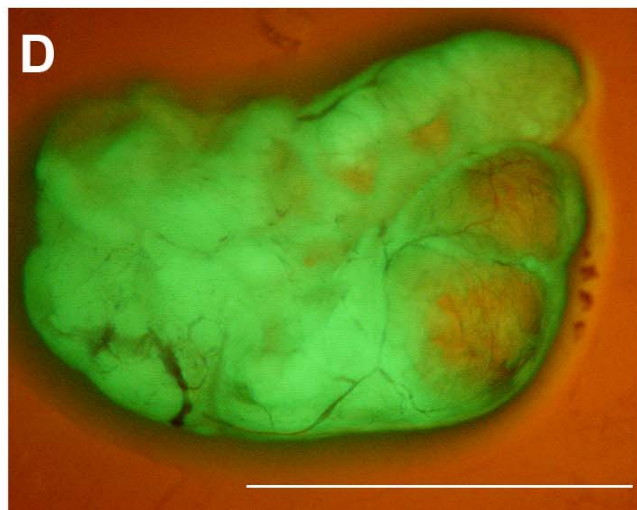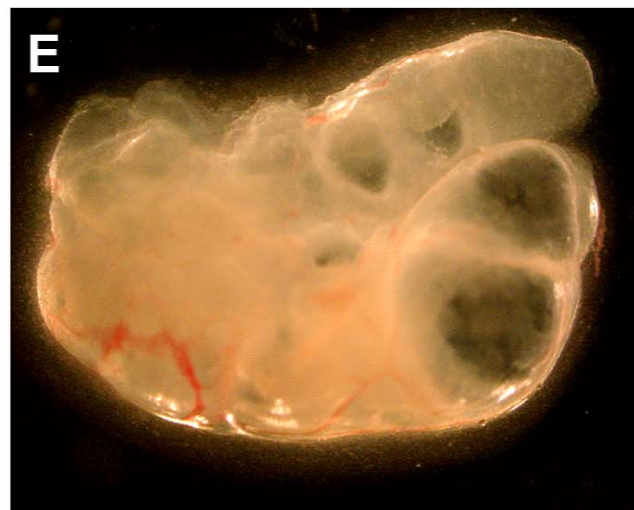

## H&E

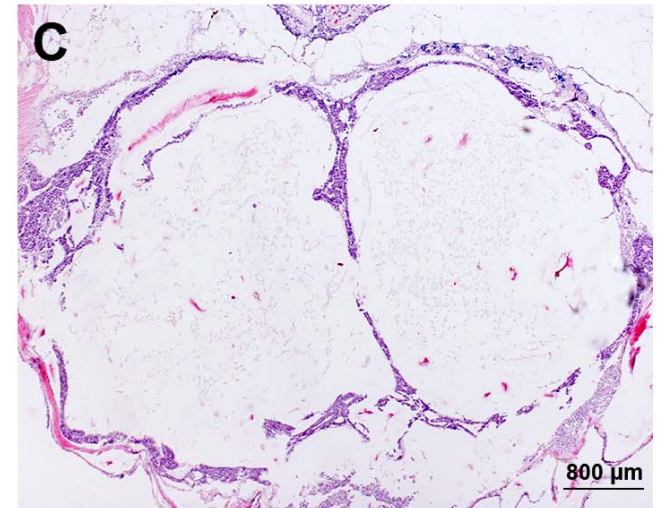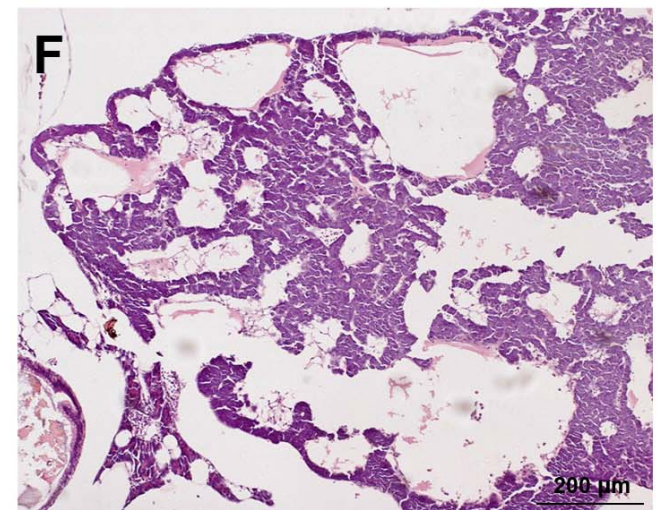

Supplement: S3 Fig — MycAG fish were induced by 2 μM mifepristone from 1 mpf and sampled at 7 mpf (6 mpi) for gross observation and histological examination. (A) Gross observation of the mycAG fish with ascites phenotype in monotone background. (B) Gross observation of the same fish in (A) under a dark background to show transparent belly. (C) H&E staining of the fish presented in (A,B). (D, E) Isolated liver tumor from another mycAG fish with ascites for GFP view (D) and for view in a dark background to show transparent, foam-like structure (E). Liquid was observed in cysts in the liver. (F) H&E staining of the liver tumor presented in (C,D). (PDF) [file pone.0117249.s003.pdf]

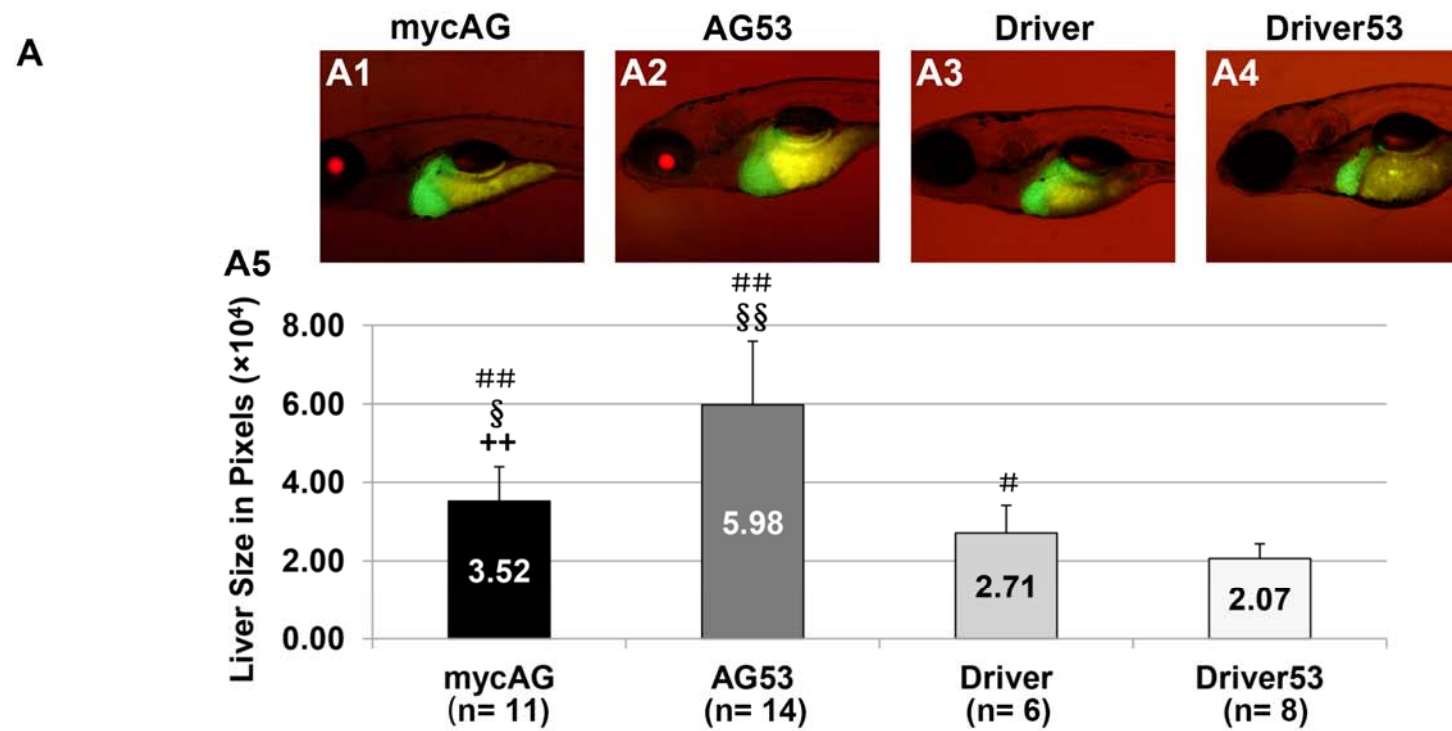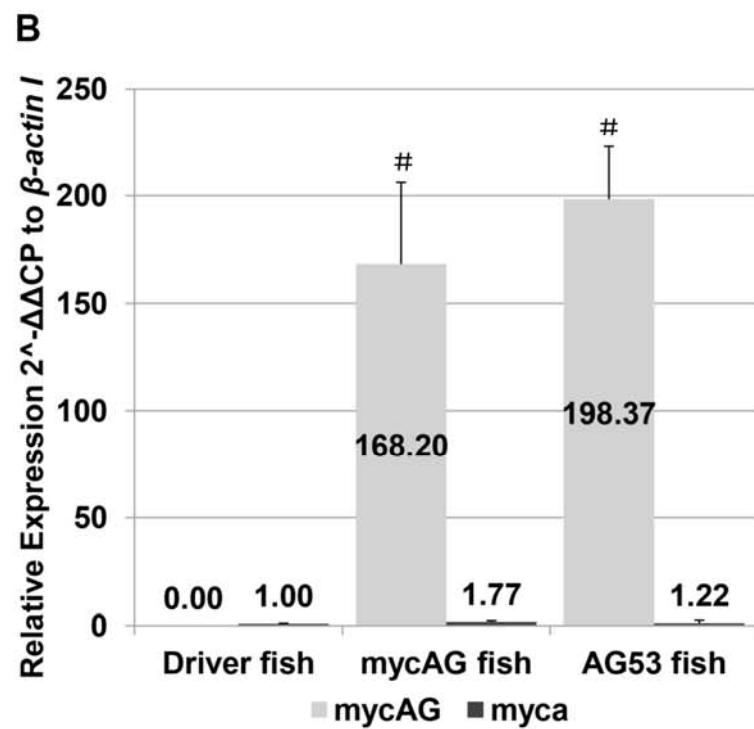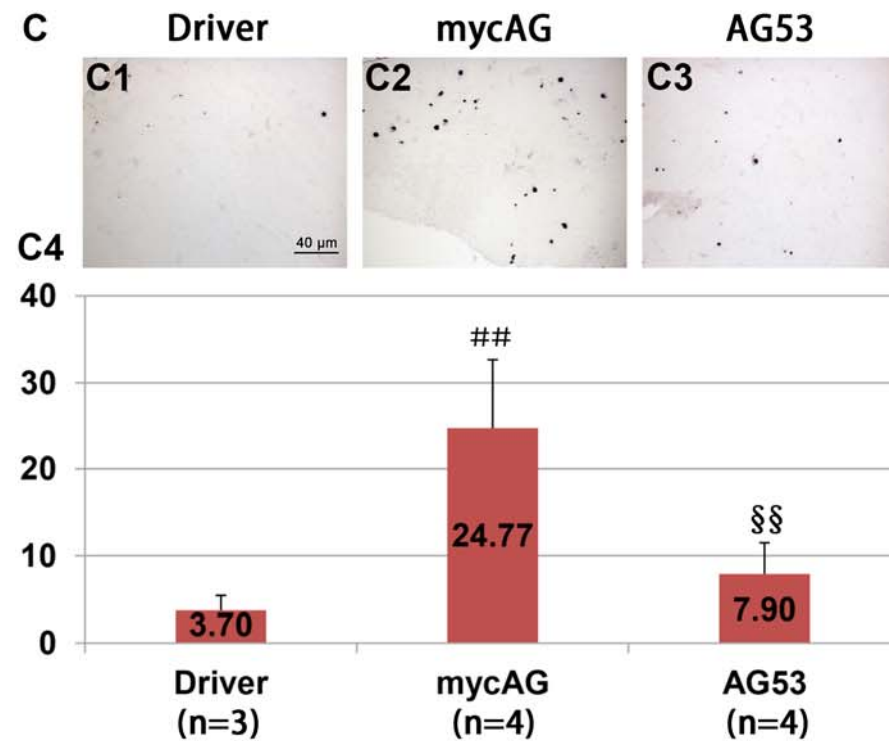

Supplement: S4 Fig — (A) Comparison of liver size between mycAG and AG53 larvae. Larvae were induced with 2 μM mifepristone from 3 dpf and photographed at 5 dpi (8 dpf). Liver size was measured based on 2D GFP images as previously described [35]. ## and # indicate significant difference with p-values of 0.01 and 0.05 respectively when compared with the liver size in the Driver53 (Driver fish in tp53M214K mutation background). §§ and § indicate significant difference with p-values of 0.01 and 0.05 respectively when compared with the liver size in the Driver. ++ indicates significant difference (P < 0.01) when compared with the liver size in AG53. (B) Induction of mycAG expression in mycAG and AG53 fish. Total RNA was extracted from 5 dpi/8 dpf larvae treated with 2 μM mifepristone for RT-qPCR analyses. Each group has three biological replicates and there was no significant effect on induction of mycAG mRNA by tp53M214K mutation. # indicates the expression differences when compared to endogenous myca expression in Driver fish (p < 0.05). (C) Apoptosis in AG53 and mycAG fish livers. Liver sections from 2 mpi (3 mpf) driver, mycAg and AG50 fish were used for TUNEL staining. Each group has 3 to 4 biological replicates and apoptosis was counted and represented in column. ＃＃, indicates the difference in proliferation when compared with Driver is significant (P < 0.01). §§ indicates significant difference between mycAG and AG50 fish (P < 0.01). (PDF) [file pone.0117249.s004.pdf]
