# Supplementary material for: Myc-Induced Liver Tumors in Transgenic Zebrafish Can Regress in tp53 Null Mutation
Source: PLoS One. 2015 Jan 22;10(1):e0117249. doi: 10.1371/journal.pone.0117249 (PMC4303426; doi:10.1371/journal.pone.0117249)
Supplement: S1 Table — (DOCX) [file pone.0117249.s005.docx]

Table S1 Primer Sequences Used in PCR

| Gene Name | Forward (5’-3’) | Reverse (5’-3’) |
| --- | --- | --- |
| *mycAG* | ACGAGCAGAGGCTGCTGTCCAT | TCAGGGTCAGCTTGCCGTAGGT |
| *mycBG* | AGGCGACGGAGTGCATAGCGA | TCGCCGGACACGCTGAACTTG |
| *myca* | CGCGCTACGGGATGAGATCCCT | GCAGGGGGTGGGAGTTCTTGGA |
| *mycb* | AAGCGGCCAAAGTGGTGATCCT | CACTACTTTGCCACACCCTCGC |
| *β-actin* | CCACCTTAAATGGCCTAGCA | CATTGTGAGGAGGGCAAAGT |
| *tp53*_wild type | AGCTGCATGGGGGGGAT | GATAGCCTAGTGCGAGCACACTCTT |
| *tp53*_mutant | AGCTGCATGGGGGGGAA | GATAGCCTAGTGCGAGCACACTCTT |
